# Habitat variations affect morphological, reproductive and some metabolic traits of Mediterranean *Centaurea glomerata* Vahl populations

**DOI:** 10.1016/j.heliyon.2020.e04173

**Published:** 2020-06-12

**Authors:** Mahmoud O. Hassan, Suzan A. Tammam, Hanaa Kamal Galal, Samir M. Saleh, Mona Sayed, Ahmed Amro

**Affiliations:** a Department of Botany and Microbiology, Faculty of Science, Beni-Suef University, Beni-Suef, E-62511, Egypt; b Department of Botany and Microbiology, Faculty of Science, Assiut University, Assiut, Egypt; c Biology Department, Faculty of Sciences and Arts, Al-Baha University, Al-Baha, KSA; d Central Laboratory for Agricultural Climate (CLAC), Agricultural Research Center (ARC), Dokki, Giza, Egypt

**Keywords:** Morphological traits, Reproductive traits, *Centaurea glomerata*, Biochemical strategy, Phenols, Flavonoids, Environmental analysis, Secondary metabolites, Systems biology, Wildlife ecology, Soil science

## Abstract

*Centaurea glomerata* Vahl is an annual, monoecious and herbaceous member of Asteraceae, found in some localities of different topographic features/habitat conditions along the Mediterranean coastal region of Egypt. This study aimed to investigate some environmental gradients including edaphic and climate criteria on morphological, reproductive traits as well as phenolic and flavonoid metabolites in this species. Three distinct populations were selected. Two of them were located in coastal sand dunes (found in Rosetta region in Egypt); one was located on flat sand dunes, whereas the other grown on sloping ones. Meanwhile, the third population was represented in the rocky hillside of Burg El Arab region. The population detected in the sloping sand dunes showed best morphological and reproductive features, whilst the opposite was true for that represented on the rocky hillside. Moreover, the free phenolic and flavonoid compounds prevailed in the later. The meteorological data revealed that the rocky hillside received relatively lower minimum temperature and higher solar irradiance, while the sand dunes of Rosetta showed more warmer conditions. Light intensity and wind speed were reduced on the sloping sand dunes. The Canonical Correspondence Analysis (CCA) exhibited a clear correlation between most of metabolites detected and the population found on the rocky hillside along with higher solar irradiance prevails. The morpho-reproductive traits were related to climatic gradients and some soil criteria. These results revealed that the changes in micro-topography, that may lead to change in soil and climate variables, is the most important environmental gradient that controls the morphological and biochemical features of *C. glomerata*. Solar irradiance and/or light intensity are key factors playing a role influencing the measured traits of this species. These findings suggest that accumulation of secondary metabolites could be a biochemical strategy and an adaptational criterion for such species under stress conditions.

## 1 Introduction

The ecological environments surrounding plant species are mostly heterogeneous, creating ecological variables that influence plant development along the prevailing environmental gradients (Bazzaz, 1996). Many ecologists have emphasized the response of a given species to these variations in environmental factors (Hegazy et al., 2010; Salama et al., 2018). Even within the same habitat, some microhabitats have high environmental variability affecting the persistence of species and demography of populations (Levine et al., 2008). The change in microtopography associated with varying edaphic and climatic factors may impose populations of some species to develop locally adaptable functional traits (Hegazy, 2001; Lobo et al., 2003). To highlight the strategy of certain plant species to counteract these ecological conditions is one of the major goals for ecologists. Several studies have revealed the significance of morphological, biochemical and reproductive traits to identify and interpret plant responses to environmental heterogeneity (Dujardin et al., 2011; Kahmen and Poschlod, 2008; Roux et al., 2017). Additionally, most studies have undertaken the allocation and partitioning of resources around different plant organs (e.g., Patty et al., 2010; Vilela et al., 2012). However, other plant species found in different habitats and/or microhabitats have not been explored yet, and studies are still lacking, particularly under arid and semi-arid conditions where water economy amongst populations is a more influential factor. In addition, the common adaptations of some traits with respect to morphology, reproduction and biochemical activities of plant populations may be more obvious under these circumstances. Therefore, it will be interesting to study additional missing plants and, consequently, fill this gap.

The secondary metabolite production process may represent an acclimation or adaptation tool through which a given plant species may cope with environmental changes either within distinct microhabitats of the same location or among specific habitats (Rahimmalek et al., 2009; Telascrea et al., 2007). Phenolics and flavonoids, in particular, are mostly common among the various secondary metabolites found in the plant kingdom. They are considered 'eco-molecules' as they show relevant variations under variant environmental conditions and are produced and accumulated as a plastic response to a wide range of environmental constraints (del Valle et al., 2015; Sampaio et al., 2016). Moreover, the protective functions of the various phenolic and flavonoid compounds against stress conditions in plants are related to their antioxidant activities (Nakabayashi et al., 2013). Roux et al. (2017) have studied the effects of these environmental factors on some physiological and phytochemical criteria of *Inula montana* in Southeastern France. However, they have only investigated total polyphenols and flavonoids with special reference to some lactones that show biochemical adaptations under certain environmental conditions. Field studies aimed at establishing a relationship between plant productivity and metabolite accumulation are few (Hofmann and Jahufer, 2011). Estimation of these metabolites, particularly free and total phenolics and flavonoids, in a certain species to develop a relationship between them and the species environment is still lacking. Thus, we investigated such possible correlations in the Mediterranean basin belonging to Egypt. In addition to abiotic components, the biotic environment has a substantial effect on the life history and physiology of some species, and these interactions may shift the metabolic responses of plants (Van De Velde et al., 2019). For example, an allelopathic interaction may affect the growth and biosynthesis of flavonoids in plants (Hassan et al., 2014; Gomaa et al., 2015). Besides, competition may result in a stronger decline of plant growth synchronizing with higher allocation of more resources towards the production of secondary metabolites (Broz et al., 2010). Ecological evaluation of these variations may be helpful in the chemical characterization of plant specimens of the same species collected from different geographical territories (Lukas et al., 2009; Stashenko et al., 2010).

Many species found in distinct habitats or microhabitats that show these responses to their habitats have not been explored yet, and studies are still lacking, particularly under arid and semi-arid conditions where water is a more influential factor. Moreover, change in soil characteristics in the Mediterranean region may drive local adaptation in plants (Terés et al., 2019). In this study, in a two-year study, we tried to reveal the effects of some ecological variables in terms of climate, soil and altitude on some morphological and biochemical traits in *Centaurea glomerata*, as an example, among specific habitats at the Mediterranean coast in Egypt. In addition, we depicted the effect of microtopography within the same habitat conditions on these traits.

*Centaurea glomerata* Vahl, belonging to Asteraceae, was recorded as one of sixteen species of the genus *Centaurea* amongst the Egyptian flora (Boulos, 1995). This species has been the subject of interest of many investigators, particularly due to its contents of flavonoids and sesquiterpene lactones (El-Masry et al., 1985; El-Toumy et al., 2011; Formisano et al., 2012). *C. glomerata* has been a focus due to its potential use in folk medicine (Bidak et al., 2015) and its antioxidant properties (El-Toumy et al., 2011). However, the detected metabolites in this species were not sufficient to generate a good profile of certain groups of metabolites. Therefore, further study may be needed to identify more phenolics and flavonoids from this plant. This species is restricted to some habitats in the Mediterranean coastal region in Egypt (Shaltout et al., 2015). It was also recorded as near endemic among endangered flora in the Mediterranean coastal region of Egypt (Ahmed et al., 2014). Due to its distribution among distinct habitats, it may show potential adaptive responses to various environmental conditions.

In light of these statements, two main hypotheses will be tested in this study: (1) as the surrounding environment poses more stressful/unfavourable conditions on growth/development of the tested species, both morphological and reproductive attributes will be adversely affected, while more resources will be allocated for the reproductive structures if compared with those allocated to the vegetative parts. In addition, both traits will be negatively correlated as a trade-off strategy. This test will confirm morpho-reproductive adaptation to the ambient conditions among the different habitats. Conversely, production/accumulation of phenolics and flavonoids, as individual compounds or total contents, is likely occurring in the more-stressed species. That is, bioaccumulation of these compounds will be negatively correlated with the vegetative attributes of this species. To detect and identify more metabolites, an adequate approach such as HPLC, with a wide range of standard compounds, could be used to obtain prospective profiles for such metabolites in plant samples and consequently correlate them with the available environmental data (climate, soil and altitude) from distinct regions. (2) Changes in topography will substantially modify plant traits due to the potential variability in altitude, soil and microclimate. Even if the altitudes are extremely convergent, the edaphic and climatic factors may be more efficient at exerting an effect. To test both hypotheses, the abovementioned traits will be estimated in plant samples collected from different populations located in different major sites (i.e., different habitats) and in two microsites if possible (i.e., within the same habitat) due to the potential variation in microtopography. Both hypotheses will confirm the 'trade-off' principle in terms of resource allocation and metabolite biosynthesis as well.

## 2 Materials and methods

### 2.1 Study area and sites of interest

The experiment was carried out in the Mediterranean coastal region of Egypt. This zone is represented by the coastal belt along the Mediterranean Sea with a width of 20–30 km southwards. The prevailing climate of this region is mainly hot dry summer and mild rainy winter, with an average annual rainfall ranging from approximately 80–150 mm. A variety of distinct habitats were recognized in this territory (Batanouny, 1973). Even within the same habitat, some microhabitats with variable vegetation characteristics were also studied (Shaltout et al., 2015). These habitats/microhabitats differ according to their microclimate and soil characteristics. Therefore, an environmental heterogeneity system is well-known in this region.

The Mediterranean sand dunes are one of the common sites that have been heavily studied due to their vegetation characteristics (Batanouny, 1999; Shaltout et al., 2015). These dunes are of an irregular topography, creating many microhabitats that support different types of plant growth. Distribution of plant communities in the northern coastal region was controlled by many factors such as topographic features (Ayyad and Ghabbour, 1986). However, so far, ecophysiological studies involving morphological, reproductive and metabolic traits of plant species in relation to topographic differences in this area are scarce.

**Figure 1:**
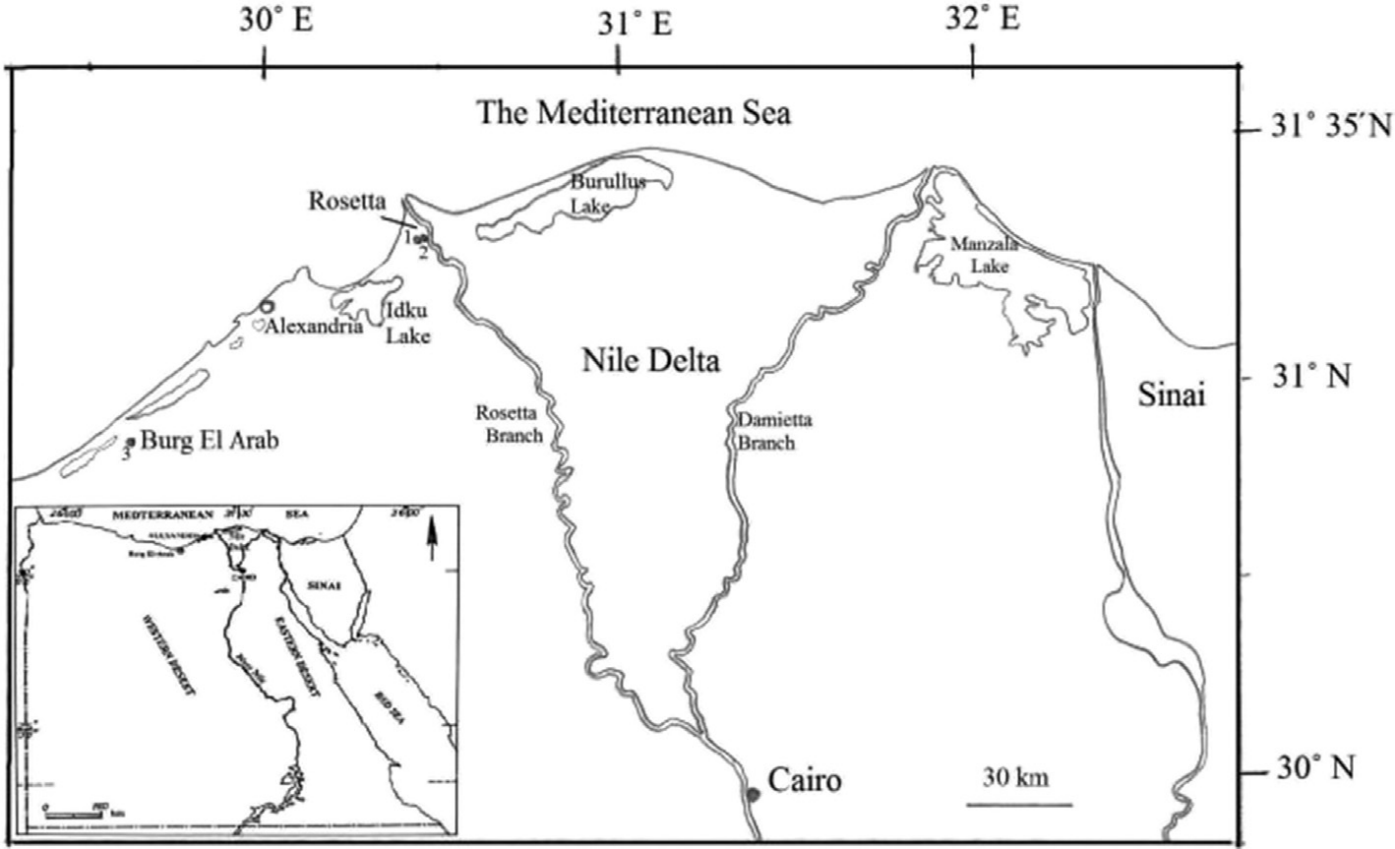
The map of the study area showing its structure, location and coordinates in Egypt. The sampling sites are represented by numbers: 1 is the plain sand dunes, 2 is the sloping sand dunes in Rosetta and 3 is the rocky hillside in Burg El Arab.

Two major locations with diverse topographical attributes in the Mediterranean coastal zone were virtually chosen to collect plant samples. These were found in two major cities in Egypt: Rosetta and Burg El Arab. Rosetta is a city in the Nile Delta, located 65 km east of Alexandria and 263 km north of Cairo. It is located at the end of the Rosetta branch (called Rashid) of the River Nile and belongs to Egypt's Beheira governorate. On the other hand, Burg El Arab is an industrial city belonging to the Alexandria governorate, Egypt. It is located approximately 45 km west of Alexandria (Figure 1). In Rosetta, two separated microsites of various topographical characters were selected. The first microhabitat was found on flat sand dunes, while the second was found on sloping ones. Both microsites were 10 m apart and approximately 7.5 km from the seashore. In Burg El Arab, the *C. glomerata* population was detected on an elevated rocky hillside. This location was approximately 105 km from the location found in Rosetta and 4.5 km from the seashore. Location coordinates and some biotic and abiotic characteristics of the microhabitats studied are well-illustrated in Table 1.

**Table 1:** Location coordinates and some abiotic characteristics recorded under field conditions on the microhabitats studied. N = North, E = East, a.s.l. = above sea level.

Parameter	Rossetta		Burg El Arab
	Flat sand dunes	Sloping sand dunes	Rocky hillsides
Latitude (N)	31° 23′ 04.30″	31° 23′ 05.10″	30° 54′ 41.60″
Longitude (E)	30° 25′ 12.90″	30° 25′ 12.90″	29° 31′ 3.70″
Elevation (m a.s.l.)	19	13	30
Density of population (No. m^−2^)	8	4	13
Wind direction	NW	NW	NW
Soil depth (cm)	30–40	˂ 90	˂ 10
Apparent soil compactness	Less compact	Very loose	Very compact with hard crust

### 2.2 Plant sampling

Plant sampling from both study sites (Rosetta and Burg El Arab) in the Mediterranean zone was carried out in mid-April and May 2017 and 2018. These periods coincided with the full flowering and fruiting stages, respectively. Additionally, pure populations were selected to avoid the effect of interspecific competition. The twelve most morphologically similar, well-developed individuals were collected from each population. The plant samples were free from missed, separated or sectioned parts, indicating the absence of predators of the above-ground parts. Furthermore, there were no holes or abnormal spots on shoots or roots of the collected specimens. The whole plants were slowly uprooted to maintain their roots. The plant samples were cleaned of suspended soil particles and loose debris.

### 2.3 Morphological and reproductive traits

Some morphological traits such as lengths of the main roots and floral stems as well as the leaf area were measured. Some plant samples (n = 6) subjected to analysis of some metabolites were freeze-dried to prevent potential degradation of the metabolites during dryness. Other plant samples (n = 8) were oven-dried for 48 h at 80 °C for potential measurement of total and fractional biomass. Each of the individuals was weighed to determine the total biomass (expressed as g individual^−1^). All plant samples were separated into roots, leaves and reproductive organs. The biomasses of below-ground roots, leaves and reproductive organs were determined. The stem mass was not included in the measured criteria as *C. glomerata* was recorded as stemless (Boulos, 1995).

The reproductive traits per individual were assessed at the peak of the flowering stage, i.e., April 2017 and 2018, and fruiting stage, i.e., May 2017 and 2018. In the laboratory, the number of heads per individual was counted. The reproductive output was evaluated by counting the number of heads and fruits (achenes) per plant. We also weighed the heads (including peduncles and the flowers with their ancillary structures) after the drying process and the mass of seeds to estimate the mean seed mass.

To estimate the sexual reproductive effort, we calculated the relative reproductive allocation (RRA) (Dujardin et al., 2011) in order to evaluate changes in strategies of biomass allocation in the species under study among habitats (Bazzaz, 1996). RRA is defined by the equation: RRA = Sexual reproduction biomass/Vegetative biomass.

### 2.4 Soil and climate characteristics

Three random soil samples (0–30 cm depth) from each habitat were collected for soil analysis. The rhizosphere soil samples collected were completely free of small snails and worms. This confirms that below-ground predators were absent. The soil samples were passed through a 2 mm sieve to remove plant residues and gravels. These samples were air-dried and stored in plastic bags until analysis. The soil texture was determined using the sieve method by calculating the amount of each fraction (gravels, sand, silt, and clay) relative to the original weight used (expressed as a percentage) (Jackson, 1967). The soil samples for each stand were oven-dried at 105 °C for 48 h. Two soil-water extracts were prepared to measure the soil chemical properties. Soil electric conductivity (EC) was measured in soil extract (1:5 w/v) by a conductivity meter (Jenway 3305), while soil pH was measured in another soil extract (1:2.5 w/v) using a digital pH meter (Hanna pH 211). Oxidizable organic carbon content (OC) was measured using the Walkley and Black rapid titration method (Black, 1979). The estimated cations and anions were measured in the former extract. Sodium amounts in the soil were identified via a flame photometer (Model Carl-Zeiss DR LANGE M7D). Soil NH_4_^+^, NO_3_^−^ and phosphate-P were measured using the method of Allen (1989). Soil NH_4_^+^ and NO_3_^−^ were extracted using 25 g of soil in 100 ml of 2 M KCl solution. After filtration, the NH_4_^+^ and NO_3_^−^ in the extract were separately measured in a spectrophotometer (UV-Vis 1601 PC; Shimadzu, Kyoto, Japan) using the indophenol blue and the cadmium reduction methods, respectively. Soil phosphates were extracted in NaHCO_3_ solution, and measurement was carried out by means of the molybdenum-antimony colorimetric method. Calcium and magnesium contents were estimated by titration against ethylenediamine dihydrogen tetraacetic acid (EDTA) using ammonium purpurate and eriochrome black T as indicators, respectively (Jackson, 1967). Soil chlorides were investigated by a direct titration process against AgNO_3_ using potassium chromate as an indicator. Soil sulphates were detected by a turbidimetric technique with the barium chloride and acidic sodium chloride solution method (Bardsley and Lancaster, 1965) using the same spectrophotometer.

The potential meteorological data of three years (2016–2018) represented by temperature (min. and max.), rainfall, wind speed, relative humidity and solar irradiance surrounding both major sites were obtained from the Agrometeorological Application Research Department (AARD), the Central Laboratory for Agricultural Climate (CLAC), Egypt. Furthermore, on the ground of each microsite, some changeable criteria were also measured, including wind speed (expressed as a percentage of the measured wind speed of the whole location) using an anemometer (Casella anemometer for measuring wind velocity, No. 4319, code SC1086, Casella London, England) and light intensity (expressed as percentage of the direct sunlight). The other measured climate parameters seemed to be unchangeable. Thus, they were not included in this study.

### 2.5 Determination of phenols and flavonoids

#### 2.5.1 Extraction of polyphenols

The polyphenols were extracted from the ground freeze-dried *C. glomerata* samples using 80% aqueous methanol in a 250 ml Erlenmeyer flask by the ultrasound-assisted method (Kim and Lee, 2002). A mixture of 10 g of plant powder and 100 ml of 80% aqueous methanol was sonicated for 60 min. The mixture was filtered, and the filtrate was then evaporated in a rotary evaporator under vacuum at 40 °C. The phenolic residue was dissolved in 50 ml of pure methanol, and the final volume of 100 ml was obtained using distilled water. The solution then was centrifuged in a Sorvall RC-5B refrigerated super-speed centrifuge at 12000 rpm for 15 min. The final extract was finally stored as a stock at -20 °C for analysis. The extraction process was carried out with six replicated plant samples.

#### 2.5.2 Determination of total phenols and flavonoids

Determination of the total phenols was performed using the protocol of Kim et al. (2003). A 1 ml portion of the stock extracts was added in a 25 ml volumetric flask filled with 9 ml of distilled H_2_O. One millilitre of Folin-Ciocalteu's phenol reagent was added to the mixture and mixed slowly. After 5 min, 10 ml of 7% Na_2_CO_3_ solution was added with slow shaking. Dilution was processed to 25 ml with distilled H_2_O and then allowed to stand for 90 min. The absorbance was monitored at 750 nm versus a prepared blank. Gallic acid was used as a standard for a calibration curve. Total phenolics in the plant samples were expressed as mg gallic acid equivalent (GAE)/g dry weight (mg g^−1^ dry plant sample).

The total flavonoids in the plant samples were determined using the method of Chun et al. (2003). One millilitre of the stock extract was added to 4 ml of distilled H_2_O in a 10 ml volumetric flask. After 5 min, 300 μl of 5% NaNO_2_ followed by 300 μl of 10% AlCl_3_ were added, and the total solution was shaken for 6 min. The remaining steps were mentioned in Hassan and Mohamed (2020).

#### 2.5.3 High-performance liquid chromatography (HPLC) analyses

Analysis was performed using an HPLC (Shimadzu chromatograph) equipped with a UV-DIODE ARRAY detector to identify flavonoids and phenolics in the samples. Such analysis (including the separation process and the different standard compounds) was fully illustrated by Hassan (2018) and Hassan and Mohamed, 2020. Concentrations of the compounds identified were expressed as μg g^−1^ dry weight using six replicated plant samples.

### 2.6 Statistical analyses

The data from the morphological, reproductive and phytochemical traits and soil criteria of *C. glomerata* populations were analysed by parametric statistics using one-way ANOVA. When the ANOVA showed significant differences, we used Tukey's test for multiple comparisons of means. To compare the climatic conditions measured in Rosetta and Burg El Arab, Student's t-test was applied. Additionally, significant differences between the climate parameters that varied with respect to the microtopography (sunlight and wind speed) in different locations were obtained using one-way ANOVA, followed by Tukey's test for multiple comparisons of means. These analyses were carried out using the SPSS Statistics software package, version 20.0 (IBM Corporation, USA). To better understand the possible correlations between the environmental variables, including the edaphic and climatic factors, that influence growth, reproductive and metabolites, the canonical correspondence analysis (CCA) ordination technique was performed by the CANOCO program (Ter Braak, 1987–1992).

## 3 Results

### 3.1 Morphological traits

The measured morphological criteria varied among the locations surveyed (Table 2). Among Rosetta microsites, the plant samples on the sloping sand dunes attained better vegetative characters in terms of root length, floral stem length, leaf area and dry matter contents when compared with those found in the flat sand dunes (Table 2). In addition, these criteria were more pronounced in individuals found on the flat sand dunes of Rosetta in comparison with those found on the rocky hillside.

**Table 2:** Mean values ± SE of some morphological and reproductive traits measured in *C. glomerata* from distinct habitats/microhabitats at the Mediterranean coastal region, Egypt. (RRA = relative reproductive allocation). Different letters for the same parameter indicate significant differences among the different locations using Tukey's test at *P* ˂ 0.05.

Parameter	Rosetta		Burg El Arab
	Flat sand dunes	Sloping sand dunes	Rocky hillside
**Morphological traits**			

Root length (cm)	17.33b ± 1.6	26.17c ± 0.61	7.25a ± 0.28
Floral stem length (cm)	6.17b ± 0.48	8.67c ± 0.71	1.48a ± 0.12
Root dry matter (g plant^−1^)	0.55b ± 0.05	1.13c ± 0.03	0.16a ± 0.01
Leaf area (cm^2^ plant^−1^)	118.10b ± 23.02	228.74c ± 16.00	39.10a ± 6.80
Leaf dry matter (g plant^−1^)	1.30b ± 0.09	4.29c ± 0.18	0.30a ± 0.02
Total dry weight (g plant^−1^) (vegetative and reproductive structures)	4.30b ± 0.30	12.12c ± 0.43	1.19a ± 0.06

**Reproductive traits**			

Number of heads plant^−1^	19.00a ± 2.53	67.17b ± 6.90	13.67a ± 0.92
Number of seeds plant^−1^	321.33b ± 27.49	469.17c ± 33.82	127.50a ± 3.49
Dry weight of heads plant^−1^	2.15b ± 0.15	5.65c ± 0.37	0.58a ± 0.04
Seed mass (g plant^−1^)	0.46b ± 0.03	0.48b ± 0.32	0.11a ± 0.01
RRA	1.41b ± 0.03	1.13a ± 0.07	1.52b ± 0.10

### 3.2 Reproductive traits

In terms of dry weights of heads in addition to seed numbers and mean seed mass per plant, the plant samples collected from the rocky hillside of Burg El Arab attained lower values compared with those located in both microsites of Rosetta (Table 2). For Rosetta, the number of heads per plant and their dry weights were higher (*P* ˂ 0.05) by approximately 253.5 and 162.8%, respectively, in the individuals found on the sloping sand dunes in comparison with the flat-dune individuals. With respect to seed output, the number of seeds produced from the sloping-dune plants was higher (*P* ˂ 0.05) by approximately 46.0% compared with that counted from the flat-dune plant samples. However, the seed mass of both locations was apparently similar. The RRA was reduced in the plant population on the slope in contrast with those detected in the other sites (Table 2).

### 3.3 Diversity of phenolics and flavonoids detected

Various phenolic and flavonoid compounds were detected in *C. glomerata* in the locations surveyed, and their contents varied among the habitats studied (Table 3). Except for p-coumaric and protocatechuic acids, all individual phenolic compounds were increased (*P* ˂ 0.05) in the plant samples found on the rocky hillsides in contrast with the remaining sites. Moreover, this population as well as that found in the flat dunes of Rosetta had higher amounts of protocatechuic acid and total phenols in comparison with the values measured for the sloping-dunes population.

**Table 3:** Mean values ± SE of the different phenolic and flavonoid compounds detected in *C. glomerata* from distinct habitats/microhabitats at the Mediterranean coastal region, Egypt. Different letters for the same compound indicate significant differences among the different locations using Tukey's test at *P* ˂ 0.05. The individual compounds were expressed as μg g^−1^ dry weight, and the total compounds were expressed as mg g^−1^ dry weight plant sample.

Compound	Rossetta		Burg El Arab
	Flat sand dunes	Sloping sand dunes	Rocky hillside
**Phenolics**			

Caffeic acid	0.72a ± 0.07	0.47a ± 0.17	1.35b ± 0.09
Chlorogenic acid	8.88a ± 0.91	10.86a ± 0.90	22.64b ± 0.91
Ellagic acid	0.12a ± 0.005	0.14a ± 0.014	0.28b ± 0.01
Ferulic acid	1.37a ± 0.39	0.95a ± 0.16	2.40b ± 0.14
Gallic acid	1.33a ± 0.07	1.70a ± 0.30	2.70b ± 0.10
p-Coumaric acid	0.15a ± 0.01	0.59a ± 0.28	0.32a ± 0.03
Protocatechuic acid	1.35b ± 0.20	0.56a ± 0.25	1.55b ± 0.13
Resorcinol	0.41a ± 0.01	0.40a ± 0.04	1.01b ± 0.04
Sinapic acid	0.80a ± 0.29	1.55a ± 0.28	3.37b ± 0.12
Syringic acid	2.17a ± 0.09	2.29a ± 0.20	4.55b ± 0.16
β-Glucogallin	0.57a ± 0.03	0.47a ± 0.11	1.25b ± 0.08
Total phenols	45.80b ± 4.45	14.95a ± 1.72	45.18b ± 3.99

**Flavonoids**			

Apigenin	0.37a ± 0.07	0.48a ± 0.078	1.01b ± 0.04
Catechin	0.012a ± 0.001	0.017a ± 0.003	0.02b ± 0.001
Daidzein	0.03a ± 0.001	0.06a ± 0.002	0.13b ± 0.005
Fesitin	0.22a ± 0.01	0.36b ± 0.014	0.85c ± 0.03
Genistein	0.21b ± 0.012	0.11a ± 0.011	0.28c ± 0.01
Isoquercetrin	0.27a ± 0.06	0.49b ± 0.031	1.05c ± 0.05
Kaempferol	22.83b ± 2.40	2.49a ± 0.35	5.38a ± 0.58
Luteolin	2.61b ± 0.17	0.19a ± 0.014	0.49a ± 0.03
Naringenin	2.34b ± 0.05	1.66a ± 0.061	4.48c ± 0.18
O-hydroxydaidzein	0.53a ± 0.06	0.62a ± 0.051	0.72a ± 0.72
Quercetin	6.56a ± 0.95	5.67a ± 0.54	6.87a ± 0.24
Rutin	0.58a ± 0.14	0.48a ± 0.045	1.15b ± 0.06
Velutin	0.63a ± 0.02	0.66a ± 0.054	1.17b ± 0.04
Total flavonoids	15.28b ± 1.02	5.87a ± 0.35	11.49b ± 0.57

Among thirteen flavonoid compounds detected, nine flavonoids accumulated (*P* ˂ 0.05) in the *C. glomerata* population found on the rocky hillside of the Burg El Arab region in comparison with both locations of Rosetta (Table 3). In contrast, the population placed in the flat dunes showed increases in kaempferol and luteolin contents in comparison with the remaining sites. Equal amounts of O-hydroxydaidzein and quercetin were observed in all populations studied. Within the Rosetta area, higher amounts of genistein and naringenin compounds were recorded in the population found on the flat dunes in comparison with those identified in the population located on the sloping dunes (by approximately 91 and 46.4%, respectively). The latter, in addition, contained lower amounts of total flavonoids in contrast with those collected from the remaining sites (Table 3).

### 3.4 Variation of soil and climate parameters

The measured physicochemical characteristics of the soil clearly varied among the habitats studied (Table 4). The soil samples in both microhabitats of Rosetta had lower contents of gravels, sand and clay. However, silt was more pronounced in soils of such area. The soil samples of Burg El Arab region were more abundant in terms of electrical conductivity, organic carbon, potassium and sulphate ingredients, whereas the opposite was correct for their phosphorus content (Table 4). As such, they manifested higher amounts of magnesium compared with the flat dunes of Rosetta. For both microsites of Rosetta region, the soil of the flat sand dunes had higher contents of gravels and sand, while it attained less amounts of clay particles compared with that found on the slope. Additionally, this microhabitat included higher values of soil organic carbon, available phosphorus, potassium and calcium.

**Table 4:** Mean values ± SE of the edaphic characteristics of the studied *C. glomerata* habitats at the Mediterranean coastal region, Egypt. Different letters for the same parameter indicate significant differences among the different locations using Tukey's test at *P* ˂ 0.05.

Soil character	Rossetta		Burg El Arab
	Flat sand dunes	Sloping sand dunes	Rocky hillside
Gravels (%)	3.68b ± 0.22	0.00a ± 0.00	15.38c ± 0.51
Sand (%)	20.96b ± 0.72	12.75a ± 0.80	26.65c ± 0.44
Silt (%)	54.38b ± 1.45	67.43c ± 0.98	29.37a ± 0.52
Clay (%)	20.41a ± 0.64	19.81a ± 0.19	28.60b ± 0.31
EC (μS cm^−1^)	142.87a ± 2.51	140.73a ± 2.18	232.67b ± 5.78
pH	8.25a ± 0.01	8.16a ± 0.02	8.15a ± 0.03
Organic carbon (%)	0.60b ± 0.01	0.38a ± 0.02	0.75c ± 0.00
NO_3_^-^ (mg g^−1^ soil)	5.56a ± 0.10	6.34ab ± 0.47	7.29b ± 0.57
NH_4_^+^ (mg g^−1^ soil)	7.37a ± 0.14	8.40ab ± 0.62	9.67b ± 0.76
P (mg g^−1^ soil)	17.02c ± 0.89	12.12b ± 0.45	9.30a ± 0.01
K (mg g^−1^ soil)	0.09b ± 0.001	0.08a ± 0.003	0.13c ± 0.005
Ca (mg g^−1^ soil)	0.43b ± 0.02	0.32a ± 0.017	0.47b ± 0.04
Mg (mg g^−1^ soil)	0.24a ± 0.03	0.34ab ± 0.06	0.42b ± 0.02
SO_4_^2-^ (mg g^−1^ soil)	6.63a ± 0.12	3.83a ± 0.62	14.33b ± 0.62
Cl (%)	0.28a ± 0.04	0.31a ± 0.05	0.25a ± 0.02
Na (mg g^−1^ soil)	0.20a ± 0.003	0.13a ± 0.01	0.25a ± 0.01

Throughout the growing season of *C. glomerata*, the climate data somewhat showed some variations from Rosetta to Burg El Arab areas (Table 5). In Rosetta, the wind speed was higher only in April by 17.74%. Additionally, the average minimum temperature was higher from October to January (*P* ˂ 0.05) and in March (*P* ˂ 0.01). For Burg El Arab area, the solar irradiance was intense over six months of the growing season in comparison with that measured in Rosetta.

**Table 5:** The average meteorological data recorded at Rosetta and Burg El Arab regions in months of the growing season of *C. glomerata*. The values inside the parentheses represent the climate data of Rossetta and those outside the parentheses represent those of Burg El Arab. The data represent the means of 3 years of records (2016–2018).

Climate parameter	Months of the growing season							
	Oct.	Nov.	Dec.	Jan.	Feb.	Mar.	Apr.	May
Rainfall (mm)	11.94 (23.45)	23.59 (22.51)	28.76 (49.24)	32.75 (25.39)	9.50 (8.56)	2.00 (2.70)	32.70 (19.21)	0.03 (0.01)
Wind speed (km hr^−1^)	3.17 (3.56)	2.83 (3.30)	3.36 (3.99)	3.41 (4.03)	2.80 (3.25)	3.25 (3.74)	3.10 (3.56)∗	3.45 (3.85)
Relative humidity (%)	62.45 (65.10)	64.46 (65.02)	67.97 (68.74)	68.10 (68.48)	65.60 (68.19)	59.85 (63.59)	55.30 (60.47)	54.00 (59.37)
Maximum temperature (˚C)	28.57 (27.74)	23.93 (23.75)	19.27 (19.41)	17.50 (17.45)	20.25 (19.65)	23.03 (22.00)	26.91 (25.40)	29.86 (28.10)
Minimum temperature (˚C)	19.31 (21.10)∗	15.63 (17.77)∗	12.22 (14.49)∗	9.44 (11.75)∗	10.18 (12.25)	11.89 (13.65)∗∗	14.14 (15.85)	17.75 (19.20)
Solar irradiance (MJ.m^−2^. day^−1^)	18.97 (17.20)∗∗	11.56 (9.79)	10.10 (8.78)	12.56 (10.50)∗∗	14.10 (12.25)∗	20.14 (17.80)∗∗	24.43 (22.30)∗∗	27.24 (25.40)∗

∗ Significant differences between both sites at *P* < 0.05 according to Student's t-test.

∗∗ Significant differences between both sites at *P* < 0.01 according to Student's t-test.

Within the same habitat, the wind speed and light intensity on the ground were also measured in each microhabitat (Table 6). For Rosetta, both climate variables were reduced on the sloping sand dunes by 21 and 26%, respectively, when compared with those measured on the flat ones.

**Table 6:** The climate criteria measured on the ground (expressed as % from the main climate data) and changed on the sloping sand dunes in comparison with the remaining habitats. The remaining climate parameters are not shown as they have not been changed in the habitats studied. Different letters for the same parameter indicate significant differences among the different locations using Tukey's test at *P* ˂ 0.05.

Climate parameter	Rossetta		Burg El Arab
	Flat dunes	Sloping dunes	Rocky hillside
Sunlight (%)	97.87b ± 3.87	76.78a ± 6.17	98.15b ± 4.09
Wind speed (%)	98.25b ± 12.01	72.25a ± 9.45	99.5b ± 10.11

### 3.5 Canonical correspondence analysis (CCA)

**Figure 2:**
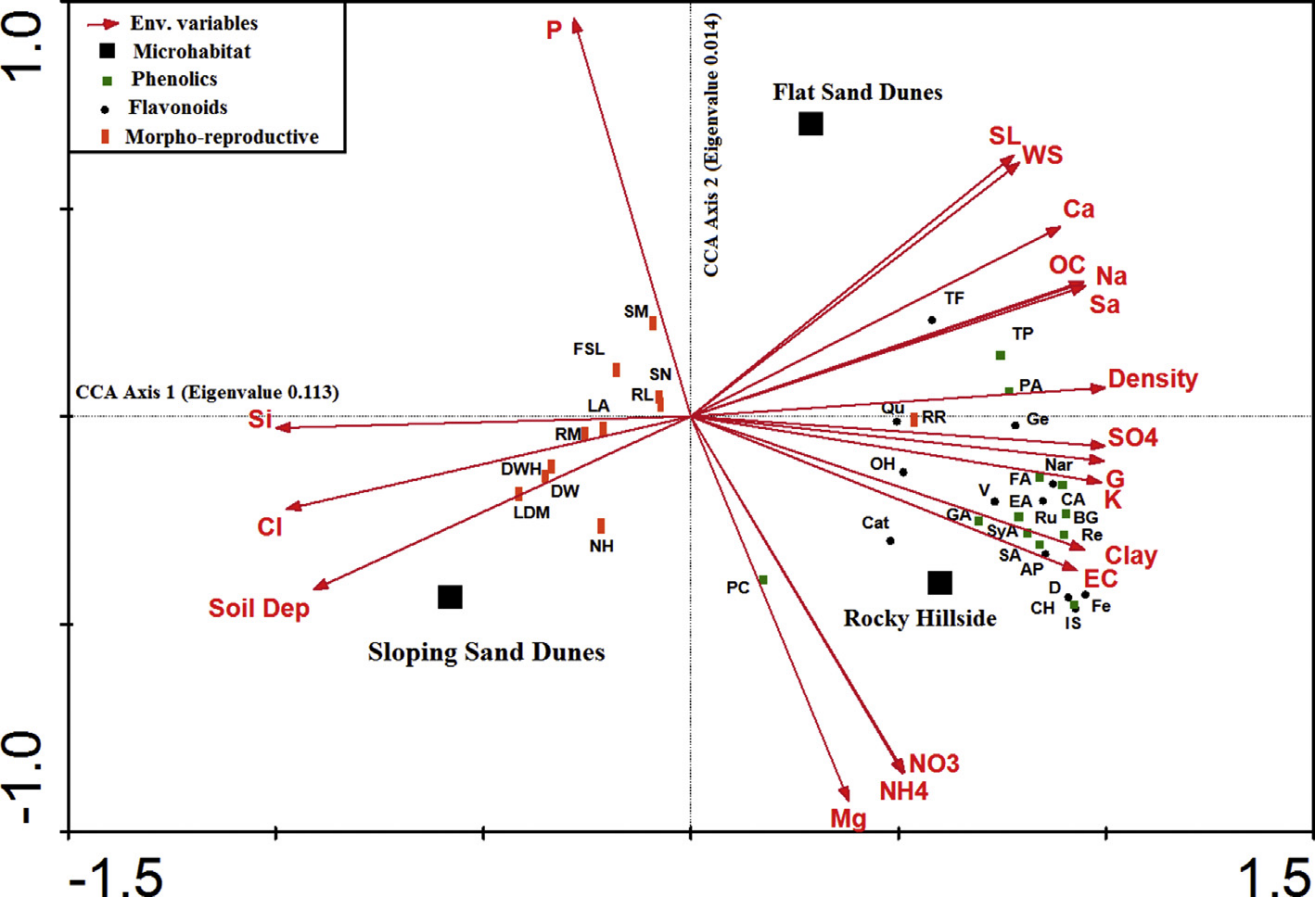
Triplot of CCA showing the possible correlations between soil variables and *Centaurea glomerata* traits. AP = Apigenin, BG = β-glucogalin, CA = Caffeic acid, Cat = Catechin, CH = Chlorogenic acid, D = Daidzain, DW = Total dry weight, DWH = Dry weight of heads, EA = Ellagic acid, FA = Ferulic acid, Fe = Fesitin, FSL = Floral stem length, GA = Galic acid, Ge = Genistein, IS = Isoquercetin, LA = Leaf area, LDM = Leaf dry matter, Nar = Naringenin, NH = Number of heads, NH_4_ = soil ammonia, NO_3_ = soil nitrates, OC = Organic carbon, OH = O-hydroxydaidzein, PA = Protocatechuic acid, PC = p-Coumaric acid, Qu = Quercetin, Re = Resorcinol, RL = Root length, RM = Root mass, RR = RRA, Ru = Rutin, Sa = Sand, Si = Silt, SA = Sinapic acid, SL = Sunlight, SM = Seed mass, SN = Seed number, SyA = Syringic acid, TF = Total flavonoids, TP = Total phenols, V = Velutin, WS = Wind speed.

The canonical correspondence analysis (CCA) ordination technique by the CANOCO program allows the identification of the best linear combinations of the environmental variables that influence the flavonoid profile in plant tissue (Figure 2). The results showed that CCA axis 1 and CCA axis 2 explained 88.7% of the variance. The Pearson's correlation coefficients between the scores of the first two axes derived from the flavonoids data and the sample scores that are linearly combined with environmental variables were 0.99 and 0.98, respectively.

Most of the investigated soil texture and cations showed high and similar factor loadings (ranging between 0.889 and 0.997) with the first axis, whereas the climatic factors (wind speed and sunlight intensity) showed a high significant loading of 0.611 and 0.629, respectively, for CCA axis 2.

This triplot also shows that the various flavonoids were segregated to vested quadrants (Figure 2). Protocatechuic acid, total phenols and total flavonoids were displaced to the upper right quadrant (plain sand dunes), and the other flavonoids and phenolic compounds were displaced to the lower right quadrant (rocky hillside). Meanwhile, all measured morphological traits of the plant were displayed in the negative scale of axis 1 and were negatively correlated with the internal secondary metabolites with sloping sand dunes.

The shoot extract profile corresponded to samples of the plants from plain sand dunes, and protocatechuic acid, total phenols and total flavonoids were the most abundant compounds. These components were highly correlated to wind speed, sunlight intensity, species density, Ca, OC and soil sand. On the other hand, most of the investigated phenolics (e.g., caffeic, chlorogenic and ferulic acids as well as β-glucogallin) and flavonoids (e.g., apigenin, catechin, daidzein, fesitin and genistein) were the most abundant components in the shoots collected from the rocky hillside and were highly correlated to soil K, Mg, NO_3_^-^, SO_4_^2-^, gravels and clay. Silt, soil depth and phosphorus were the most important environmental gradients that control the morphology and productivity of *C. glomerata* in the sloping sand dunes, which was the most suitable microhabitat where these traits were improved.

## 4 Discussion

### 4.1 Morphological and reproductive traits

Both morphological and reproductive traits of *Centaurea glomerata* are modified among the major and within the same habitats studied. The overall morphological criteria of the plant samples collected from Rosetta were promoted in comparison with those collected from Burg El Arab. In accordance with the soil characteristics of Burg El Arab, more organic matter and available nutrients such as nitrogen, potassium and calcium were substantially measured. Therefore, it is difficult to claim that the advanced morphological features of plants in Rosetta were attributed to soil resources. In addition, some soil criteria such as organic carbon, phosphorus, potassium and calcium were higher in the flat dunes. However, growth and reproductive criteria were higher for the population inhabiting the sloping sand dunes. On the other hand, our data revealed that the locations selected manifested extreme convergence in elevation. Thus, the altitudinal criterion also failed to explain such morphological differences among the different locations selected.

Variations in foliar traits refer to the different responses of plant species to variable ecological conditions (Dujardin et al., 2011; Roux et al., 2017). The results of this investigation showed that the leaf area and leaf dry mass of the plant populations found in Rosetta were remarkably higher than those measured in Burg El Arab. The climate data in this study demonstrated more warm conditions in the former (in terms of minimum temperature) and more solar irradiance in the latter. By the beginning of the growing season, the average minimum temperature was relatively higher in Rosetta from October to January, creating more favourable conditions for growth. In this regard, it was reported that warmer conditions enhance leaf growth (Åström et al., 2015; Roux et al., 2017). The increase in leaf dry mass and leaf area suggests that plants detected in Rosetta develop responses to the lower intensity of sunlight. By producing larger leaves, plants may improve the uptake of light and enhance their ability for growth. Moreover, leaf plasticity is critical for the potential adaptation of plants to specific light conditions in the Mediterranean area (Guidi and Calatayud, 2014). This result was consistent with other previous studies in this respect (Dujardin et al., 2011; Roux et al., 2017). Likewise, this behaviour could be supported by plants observed in the population located on the sloping sand dunes receiving lower light intensity in comparison with those found on the neighbouring flat dunes. Therefore, microtopography in Rosetta could have an effect on the morphological traits of *C. glomerata*. In our study, the gradual root growth among the habitats clearly followed the order: slope ˃ flat dunes ˃ rocky hillside. In addition to the effect of lower light intensity in Rosetta, the loose soil on the sloping sand dunes may create good circumstances for better allocation of resources to roots and, consequently, root growth. Conversely, the shallow soil and hard crust of the rocky hillside may hinder root extension. The heavy root growth could facilitate absorption of water and minerals, leading to better growth.

In terms of the dry weights of heads, number of seeds and average seed mass produced in each plant, *Centaurea* populations recorded in Rosetta attained higher values compared with those detected in Burg El Arab. This result suggests that reproductive allocation was substantially greater in the Rosetta region. On the other hand, the population found on the sloping sand dunes had more floral heads and lower RRA in contrast with the other sites surveyed. These results indicate that light intensity plays a fundamental role in such responses. Additionally, Yaqoob and Nawchoo (2017) showed that plants growing in shady habitats are more fit and produce larger numbers of inflorescences in contrast to those growing in direct sunlight. Moreover, in a two-year study, Jacquemyn et al. (2010) proved that decreased light conditions were more favourable for flowering and, thus, concluded that the cost of flowering was greater in shaded populations.

### 4.2 Phenolics and flavonoids

Phenolics and flavonoids have been widely proposed as developmental regulators and/or signalling molecules in plants exposed to a wide range of environmental stimuli (Pollastri and Tattini, 2011; Shalaby and Horwitz, 2015). Our data showed that most of the free phenolics and flavonoids were recorded in plants from the rocky hillside of Burg El Arab. In addition, their total contents substantially accumulated in all plant populations except for that found on the sloping sand dunes. This result followed the strong solar radiation measured in Burg El Arab. This finding also suggests the photoprotective role of polyphenols and flavonoids (Agati and Tattini, 2010; Zoratti et al., 2014). Accumulation of polyphenols has been reported in *Thymus vulgaris* under direct sunlight in comparison with shady conditions (Zrig et al., 2016). Additionally, flavonoid accumulation was reported in *Silene littorea* populations under higher solar radiation (del Valle et al., 2015). On the other hand, the equal amounts of total phenolics and flavonoids in the rocky hillside of Burg El Arab and in the flat dunes of Rosetta imply that accumulation of these compounds may be related to duration of light rather than its intensity.

For further scrutiny, the results revealed that the amount of free flavonoids was higher in the flat dunes of Rosetta. This increase could be attributed to the higher contents of kaempferol in the plain sand dunes of Rosetta. However, the number of free flavonoids was higher in Burg El Arab. In addition, more kinds and contents of free phenolics were also observed in the latter. This result indicates that the photoprotective role of flavonoids may be related to the number of free phenolics rather than their quantity. Simultaneously, the higher number and quantity of free phenolics may have another protective role for the *C. glomerata* population in Burg El Arab.

### 4.3 Canonical correspondence analysis (CCA)

It is clear that the flavonoid profiles of plants collected from the sunny rocky hillsides clearly differed from those grown on shaded sloping sand dunes. Meanwhile, a negative correlation was established between morpho-reproductive criteria and the accumulated metabolites. This result suggests that these compounds play an essential role as an adaptational response to an opposing environment, showing that an investment in plant metabolites increases simultaneously with growth limitations (Mooney, 1991). According to the results obtained in this work, it is possible that sun-exposed environments trigger the production of phenolics and flavonoids. According to such analysis, most free phenolics and flavonoids were closely correlated with some soil characters including soil potassium, sulphates, clay and gravels. Therefore, these parameters might induce these compounds due to better nutrition. However, we cannot completely rely on these items as factors. Some authors have used flavonoids as speciation markers (Reynaud and Lussignol, 2005). Kade et al. (1997) suggested that these compounds might be important markers for distinguishing populations or ecotypes. Some of them, particularly total compounds, were relatively related to sunlight and wind speed. Plant secondary metabolites can be gradually synthesized in response to harsh environmental factors (Yang et al., 2018), and this phenomenon can be considered a plant behaviour for adaptation and survival in response to environmental stimuli during the plant life history (Metlen et al., 2009). Plants are also able to adapt to changes in light radiation by the production and accumulation of various secondary metabolites (i.e., phenolics and flavonoids), which have well-known antioxidant properties (Yang et al., 2018). For instance, *Ipomoea batatas* and *Vaccinium myrtillus* increased their catechin contents under long-day light conditions (Carvalho et al., 2010). Additionally, *V. myrtillus* increased its chlorogenic acid under full sunlight conditions (Alqahtani et al., 2015). On the other hand, CCA analysis indicated a close correlation between some detected metabolites and the population density. Moreover, the density of the population was the opposite of the morpho-reproductive attributes. This result suggests that *C. glomerata* may exhibit specificity in its response to conspecific neighbours. This result was also consistent with that obtained by Broz et al. (2010), who proved that synthesis of secondary metabolites, particularly phenolics, was induced due to conspecific competition amongst *C. maculosa* individuals. Therefore, conspecific competition may elicit responses for *C. glomerata* in terms of its morphological, reproductive and metabolic traits.

## 5 Conclusion

The present study showed substantial variability in morphological and reproductive traits of *C. glomerata* among different populations found in different locations in the Mediterranean coastal zone in Egypt. In addition, the measured phenolic and flavonoid compounds were varied. Generally, in Rosetta populations, the best growth and reproductive attributes could be attributed to the shading conditions under which the population on the sloping sand dunes grows. In contrast, this investigation shows that growing habitats inducing plant stress, particularly higher light intensity and solar irradiance, can induce the production of phenolics and flavonoids in such species. In addition, the variations exhibited in *C. glomerata* individuals may be attributed to the density of the populations studied. Higher density may result in conspecific competition, the biotic stress that reduces growth and reproductive criteria but, consequently, mediates the biosynthesis of secondary metabolites as an adaptive response. The soil criteria may have but a minor role in the obtained variation with respect to vegetative, reproductive and metabolite traits. The morpho-reproductive attributes and accumulation of free and total phenolics and flavonoids were negatively correlated. These findings suggest that accumulation of secondary metabolites could be a biochemical strategy and an adaptational criterion for Mediterranean *C. glomerata* under some stress conditions.

## Declarations

### Author contribution statement

Mahmoud O. Hassan: Conceived and designed the experiments; Performed the experiments; Analyzed and interpreted the data; Contributed reagents, materials, analysis tools or data, Wrote the paper.

Suzan A. Tammam, Hanaa Kamal Galal, Samir M. Saleh, Mona Sayed: Contributed reagents, materials, analysis tools or data.

Ahmed Amro: Performed the experiments; Analyzed and interpreted the data; Contributed reagents, materials, analysis tools or data; Wrote the paper.

### Funding statement

This research did not receive any specific grant from funding agencies in the public, commercial, or not-for-profit sectors.

### Competing interest statement

The authors declare no conflict of interest.

### Additional information

No additional information is available for this paper.
